# A Novel tiRNA-Gly-GCC-1 Promotes Progression of Urothelial Bladder Carcinoma and Directly Targets TLR4

**DOI:** 10.3390/cancers14194555

**Published:** 2022-09-20

**Authors:** Chuan Qin, Zheng-Hao Chen, Rui Cao, Ming-Jun Shi, Ye Tian

**Affiliations:** Department of Urology, Beijing Friendship Hospital, Capital Medical University, Beijing 100032, China

**Keywords:** tRNA-derived small RNA, bladder cancer, TLR4, cancer progression

## Abstract

**Simple Summary:**

Patients with urothelial bladder carcinoma (UBC) have a poor prognosis and a high risk of progression. Recently, tRNA-derived small RNAs (tsRNAs), a novel type of noncoding RNA, have been identified. In our previous study, we found differential expression profiles of tsRNAs in UBC. As a result, tiRNA-Gly-GCC-1 was significantly upregulated in UBC tissue and might target the predicted target gene toll-like receptor 4 (TLR4) to play a regulatory role in UBC. Here, after lentiviral transfection in UBC cell lines, the results showed down-regulation of tiRNA-Gly-GCC-1 could inhibit cell proliferation, migration and invasion, promote cell apoptosis, and affect the cell cycle. Besides, tiRNA-Gly-GCC-1 was found to inhibit TLR4 expression by directly targeting its 3′UTR. In summary, our study demonstrated that tiRNA-Gly-GCC-1 promotes the progression of UBC and directly targets TLR4. This study provides novel insights for future investigations to explore the mechanisms and therapeutic targets for UBC.

**Abstract:**

Background: Patients with urothelial bladder carcinoma (UBC) have a poor prognosis and a high risk of progression. Recently, tRNA-derived small RNAs (tsRNAs), a novel type of noncoding RNA, have been identified. In our previous study, we found tiRNA-Gly-GCC-1 was significantly upregulated in UBC tissue and might target the predicted target gene toll-like receptor 4 (TLR4) to play a regulatory role in UBC. Thus, the aim of this study was to identify the functional roles of tiRNA-Gly-GCC-1 and the relationship between tiRNA-Gly-GCC-1 and TLR4. Methods: After lentiviral transfection in 5637 and T24 cell lines, quantitative reverse transcription-PCR, Cell Counting Kit-8, IncuCyte ZOOM™ live cell imaging, flow cytometry, Transwell assays, scratch assay, and luciferase assay were performed. Results: The results showed down-regulation of tiRNA-Gly-GCC-1 inhibits cell proliferation, migration and invasion, promotes cell apoptosis, and affects the cell cycle. Besides, tiRNA-Gly-GCC-1 was found to inhibit TLR4 expression by directly targeting its 3′UTR. Conclusions: Our study demonstrated that tiRNA-Gly-GCC-1 promotes the progression of UBC and directly targets TLR4. This study provides novel insights for future investigations to explore the mechanisms and therapeutic targets for UBC.

## 1. Introduction

According to the data, bladder cancer (BC) has ranked 6th in the prevalence and 13th in the mortality rate of malignant tumors worldwide [1]. In 2015, the morbidity rate of BC in China was 5.80 per 100,000, ranking 13th among malignancies of the whole body, and the mortality rate of BC in China was 2.37 per 100,000, ranking 11th [2]. The pathological types of BC include the common uroepithelial, squamous cell, and adenocarcinomas, as well as the less common small cell, mixed carcinosarcoma, and metastatic carcinomas. Among them, urothelial bladder carcinoma (UBC), previously often described as transitional cell cancer, is the predominant histological type (more than 90%). According to the degree of muscle invasion, UBC is classified as non-muscle-invasive bladder cancer (NMIBC) and muscle-invasive bladder cancer (MIBC) [3]. NMIBC has a high risk of tumor recurrence (approximately 50–70%) and progression (approximately 15%) which is unaggressive and usually noninvasive. The treatment for NMIBC is usually transurethral resection of bladder tumors (TURBT), supplemented by postoperative bladder irrigation chemotherapy or immunotherapy. Compared to NMIBC, MIBC usually leads to a poor prognosis and a high risk of tumor metastasis which is far more life-threatening [4]. Notably, approximately 50% of patients μLtimately develop the disease at distant sites because of disseminated micrometastases. The treatment for MIBC includes radical cystectomy (RC) and pelvic lymph node dissection with systemic adjuvant chemotherapy. Therefore, UBC aggravates patients’ quality of life and it is one of the biggest challenges for basic science and clinical research to inhibit the progression and expand the treatment armamentarium of UBC. Besides, the diagnosis of UBC and lifelong surveillance strategies, are mainly relied on cystoscopy before surgery, an invasive intervention, which adversely affects patients’ quality of life and results in a high economic burden on healthcare systems. Thus, more precise diagnosis methods and molecular markers are needed for clinics. Many intrinsic and extrinsic genetic factors could affect the occurrence and progression of UBC [5]. The progression of UBC is regulated by a mμLti-factor network, involving the whole process of gene transcription and expression [6]. Thus, it is necessary to clarify the molecular mechanisms associated with UBC progression from different perspectives.

The majority of the human genome is non-coding RNAs (ncRNAs), which are involved in the regulation of gene expression through post-transcriptional levels and epigenetic modalities [7]. Transfer RNAs (tRNAs) are the first non-coding RNAs to be identified and the most abundant short-stranded ncRNAs [8]. Similar to other ncRNAs, tRNAs undergo a specific maturation process whereby longer primary transcripts are specifically cleaved by nucleases to produce many different classes of small ncRNAs called tRNA-derived small RNAs (tsRNAs) [9]. Depending on the splice site and length, tsRNAs can be divided into: stress-induced tRNA-derived RNAs (tiRNAs) with a length of 30–40 nts and tRNA-derived fragments (tRFs) with a length of 14–36 nts [10]. tsRNAs have similar properties to microRNAs (miRNAs), namely, they play a major role in RNA silencing through binding AGO proteins. However, there are several differences between tsRNAs and microRNAs (miRNAs): (i) tsRNAs biosynthesis process is more complex; (ii) tsRNA-mRNA binding is more extensive; (iii)tsRNAs structure is more complex and RNA modification is more extensive; (iv) tsRNAs are associated with AGO protein 1–4 while AGO2 is the main effector protein of miRNAs. In recent times, plenty of evidence has suggested that tsRNA is not merely a “byproduct” of random tRNA splicing but acts as a regμLator and plays a dynamic role in the progression of many diseases. For example, tRF5-Glu promoted the target gene expression and proliferation in ovarian cancer cells [11]. The tRF-3019a was reported to modulate gastric cancer cell proliferation, migration and invasion by targeting FBXO47, and it may serve as a potential diagnostic biomarker for gastric cancer [12]. In another case, tRF-3001b may aggravate the development of nonalcoholic fatty liver disease by inhibiting autophagy via targeting Prkaa1 [13]. In order to clarify that specific tsRNAs were associated with the pathophysiological changes in UBC, we previously used RNA sequencing, bioinformatics, and quantitative reverse transcription-PCR (qRT-PCR) to screen the expression profiles and predict the potential roles of tsRNAs in UBC. The results showed that tiRNA-Gly-GCC-1, one of four candidate tsRNAs, was significantly upregulated in UBC tissue and might target the predicted target gene toll-like receptor 4 (TLR4) to play a regulatory role in UBC [7]. For tsRNAs’ subclasses in UBC, there were a total of 13 tRF-1, 1 tRF-2, 70 tRF-3a, 21 tRF-3b, 22 tRF-5a, 13 tRF-5b, 77 tRF-5c, 2 tiRNA-3, and 26 tiRNA-5 which were significantly dysregulated after sequencing and screening. The target gene, tiRNA-Gly-GCC-1, belongs to the tiRNA-5 subclass [7]. To date, only one study of tiRNA-Gly-GCC-1 in another cancer was reported which demonstrated that tiRNA-Gly-GCC-1, upregulated in hypoxia-treated triple-negative breast cancer cell lines, might act as a regulatory factor involved in the hypoxia-induced chemoresistance [14]. However, the functions of tiRNA-Gly-GCC-1 and the relationship between tiRNA-Gly-GCC-1 and TLR4 in UBC remain unclear.

Therefore, in the present study, we sought to explore the biological functions of tiRNA-Gly-GCC-1 in UBC cell lines and identify the direct binding relationship between tiRNA-Gly-GCC-1 and TLR4. These findings were expected to provide a new theoretical basis for clarifying the molecular mechanism of UBC progression and offer a new treatment strategy for UBC in the future.

## 2. Materials and Methods

### 2.1. UBC Tissue Collection

Eight pairs of UBC and matched paracancerous tissues (with a distance of 3 cm from the tumor) were achieved from eight MIBC patients undergoing surgery at the Department of Urology of Beijing Friendship Hospital affiliated with Capital Medical University (Beijing, China) in 2021. The inclusion criteria were consistent with our previous study [7]. This study was approved by the Ethics Committee of Beijing Friendship Hospital affiliated with Capital Medical University and complied with the Declaration of Helsinki ethical standards. Tissue samples were only collected from patients when informed consent was obtained. Tissue samples were dissected during the surgery and immediately stored in liquid nitrogen. All tissues (tumor and paracancerous tissues) were used for qRT-PCR.

### 2.2. Cell culture and Transfection

In brief, human UBC cell lines (T24 and 5637) were purchased from the Shanghai Institutes for Biological Sciences, China Academy of Science (Shanghai, China). The cells were cultured in DμLbecco’s Modified Eagle’s Medium (DMEM) containing 10% heat-inactivated fetal bovine serum (FBS; Thermo Fisher Scientific, Inc., Shanghai, China), penicillin (100 U/mL), and streptomycin (100 μg/mL) at 37 °C in a 5% CO_2_ incubator.

We used lentiviral particles (Shanghai Genechem Co., Ltd.) to knock down tiRNA-Gly-GCC-1. Notably, the vector carried a green fluorescent protein (GFP) element. The short hairpin RNA (shRNA) was designed and used with target sequence (5′-CAGGCGAGAATTCTACCACTGAACCACCCATGC-3′). The sequence of negative control shRNA was 5′-TTCTCCGAACGTGTCACGT-3′. All sequences are shown in Table S1. Cell transduction was firstly performed by plating 5 × 10^4^ cells/mL in different cell plates with complete medium. After cell incubation at 37 °C for 24 h, 20–30% confluent of UBC cells was achieved. Then, cells were infected with 1–20 μL of the appropriate lentivirus (1 × 10^8^ infectious units/mL, MOI = 10) and 4–40 μL polybrene in complete media supplemented in different cell plates. After 12 h, the fresh complete culture medium was changed and cells were cultured at different time points for the next phenotypic experiments. To make visual observation of GFP-labeled fluorescence in the transfection group and control group, two videos for dynamic observation starting from 24 h post-transfection (day 0) were shown.

### 2.3. qRT-PCR

In order to clarify the expression level of tiRNA-Gly-GCC-1 and TLR4, SYBR-based RT-qPCR was conducted in tissues and cell lines. In brief, total RNA was collected from 6-well plates (3 wells per group) and 6 pairs of tissues (tumor and paracancerous tissues) using Trizol. After RNAs’ concentration and purity and integrity were verified by NanoDrop ND-1000 and gel electrophoresis, RNA pre-treatments including 3’ terminal deacetylation and demethylation were performed for tiRNA-Gly-GCC-1 using the rtStar™ tRF&tiRNA Pretreatment Kit (Cat# AS-FS-005, Arraystar). Next, complementary DNA (cDNA) was synthesized using the rtStar™ First-Strand cDNA Synthesis Kit (3′ and 5′ adaptors) (Cat# AS-FS-003, Arraystar). qRT-PCR was performed in the QuantStudio™ 5 Real-time PCR System (Applied Biosystems) with a 2× PCR master mix (AS-MR-006-5). All indicators were carried out according to the following procedure: 95 °C denaturation (10 min), 95 °C (10 s), and 60 °C (60 s), followed by 40 cycles. After the amplification reaction, the melting curve procedure was performed. The 2^−ΔΔCT^ method was used. Glyceraldehyde-3-phosphate dehydrogenase (GAPDH) was used for the endogenous control gene. All reactions were performed in triplicate.

### 2.4. Cell Proliferation and Confluence

The cell confluence and GFP-labeled fluorescence were observed using the IncuCyte ZOOM™ live cell imaging system (Essen BioScience, Ann Arbor, MI, USA). This live content imaging system could document and understand cell growth, cell behavior, and cell morphology. Parameter settings were phase and green fluorescence. Automatic filming of cell plates was conducted every 8 h. In short, starting from 24 h post-transfection, dynamic observation of cells (T24 and 5637) was performed. The cell confluence of three cell groups (transfection group, control group, and blank group) in 96-well plates was recorded and calculated every 8 h. The curve graph of phase object confluence (%) was drawn. Each group had five duplicate wells.

Cell proliferation assay was detected using Cell Counting Kit-8 (CCK-8, C0041, Beyotime, Shanghai, China). Briefly, 4 × 10^3^ cells were plated in 96-well culture plates for three cell groups (transfection group, negative control group, and blank group). In addition, each group had five duplicate wells. At 48 h, 72 h, and 96 h post-transfection, cells were incubated with 10 μL CCK-8 solution at 37 °C for 1 h, respectively. Then, the absorbance at 450 nm for each group was recorded using a microplate reader (Thermo Fisher). Cell growth curves were drawn for each group.

### 2.5. Cell Cycle and Apoptosis Assay

In terms of the cell cycle, the intracellular DNA content was detected by flow cytometric PI staining. In short, adherent cells were collected by trypsin digestion (5 × 10^6^ cells). Next, we added 200 μL of cell cycle rapid test reagent and mix gently to make a single cell suspension. Based on the manufacturer’s protocol, cell fluorescence was detected and evaluated using a FACS caliber flow cytometer and BD Cell Quest software (BD Biosciences). In terms of cell apoptosis, cells were washed twice with pre-cooled saline at 4 °C, then resuspended with 500 μL binding buffer and adjusted to a concentration of 10^7^/mL. Next, cells were stained with 5 μL annexin V-APC and 7-AAD, then incubated for 15 min at room temperature. Flow cytometry was performed and analyzed with LSRFortessa (BD Biosciences). All experiments were repeated three times.

### 2.6. Cell Invasion and Migration

In short, 5 × 10^4^ cells were plated in the upper chamber of Transwells (Corning) with an appropriate amount of medium without FBS, while the bottom chamber was added with complete medium. After 24 h and 37 °C incubation, cells migrating to the lower chamber were collected and fixed, stained with crystal violet, and counted under a microscope (Leica DMI300B) at ×200 magnification in order to assess the migration. Transwell invasion assays were carried out using Matrigel (BD Biosciences) following a similar protocol as described above (n = 4 per group).

### 2.7. Scratch Assay

We performed a monolayer scratch assay using the IncuCyte ZOOM™ live cell imaging system (Essen BioScience, Ann Arbor, MI, USA). The relative wound density is the ratio of the occupied area to the total area of the initial scratched region. In short, after the confluence of transfected cells reached 80–90% in 6-well plates, the confluent monolayer was scratched with a 1 mL pipette tip. After creating the scratch, the wells were washed twice with fresh medium to remove any cells from the scratched area and the medium without FBS was added. Then, the plate was placed into the IncuCyte ZOOM™ apparatus and images of the collective cell spreading were recorded every 24 h for a total duration of 3 days. All experiments were repeated three times. ImageJ software (Media Cybernetics) was used to analyze the migration abilities. Two videos for dynamic observation of green fluorescence were shown.

### 2.8. Dual Luciferase Reporter Gene Assay

In short, referring to the bioinformatics analysis in our previous study [7], the dual luciferase vectors TLR4-WT-psiCHECK 2 and TLR4-MUT-psiCHECK 2 were constructed by using plasmid psiCHECK 2 as the skeleton vector. Moreover, the target gene TLR4 was inserted according to the manufacturer’s instructions (Promega). Mutant TLR4 3′UTR luciferase vectors were produced in the predicted tiRNA-Gly-GCC-1-binding regions. These plasmids and a tiRNA-Gly-GCC-1 mimic were co-transfected into HEK293T cells. After 48 h of incubation, cells in all groups were collected for the firefly and renilla luciferase assay using a dual luciferase reporter gene assay kit (E1910, Promega) and a microplate reader (Lux-T020, BLT).

### 2.9. Statistical Analysis

Statistical analysis and graph preparation were performed using SPSS software (version 21.0, Chicago, IL, USA) and GraphPad Prism (version 8.2.1.441). All experiments were performed at least three times. The data were shown as the mean ± standard error of the mean (SEM). The data passed the Shapiro–Wilk (W) normality test and equal variance test. Student’s *t*-test (paired, two-tailed) was used to analyze the significance of differences between the two groups. One-way and two-way ANOVA were used to analyze the significance of differences between the three groups. *p* < 0.05 was considered statistically significant.

## 3. Results

### 3.1. Downregulation of tiRNA-Gly-GCC-1 after Lentivirus Transfection

**Figure 1:**
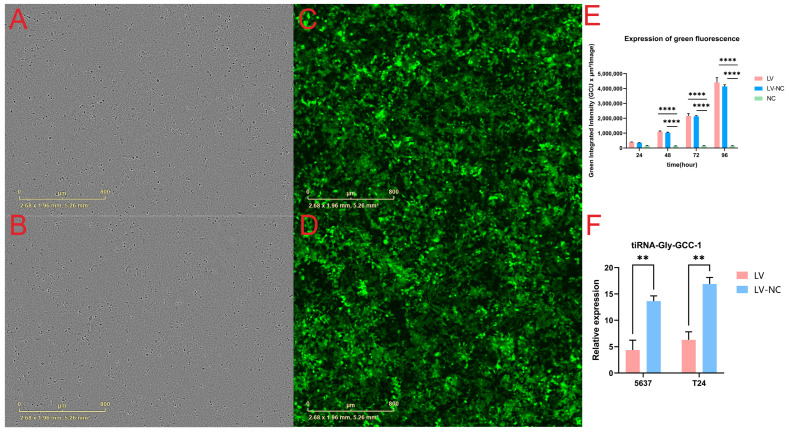
The expression of green fluorescence and down-regulation of tiRNA-Gly-GCC-1 after lentivirus transfection (transfection group, LV; negative control group, LV-NC; blank group, NC). The bright field of cells in LV group (**A**) and LV-NC group (**B**) at 96 h post-transfection in 96-well plates. The GFP-tagged lentiviral plasmids in LV group (**C**) and LV-NC group (**D**) at 96 h post-transfection in 96-well plates. (**E**) The total green object integrated intensity (GCU × µm^2^/image) in three cell groups (n = 5 wells per group). (**F**) Relative expression of tiRNA-Gly-GCC-1 of T24 and 5637 cells in LV and LV-NC group (n = 3 wells per group). The data were normalized using the mean ± SEM. ** indicates *p*-value < 0.01. **** indicates *p*-value < 0.0001.

Before the beginning of the functional experiments, we observed and detected the expression of green fluorescence using the IncuCyte ZOOM™ live cell imaging system in UBC cell lines at 24 h, 48 h, 72 h, and 96 h. As a result, 5637 cells were used as an example, Figure 1A,C shows the bright field and transfection of GFP-tagged lentiviral plasmids in the transfection group at 96 h post-transfection in 96-well plates. Figure 1B,D shows the bright field and transfection of GFP-tagged lentiviral plasmids in the negative control group at 96 h post-transfection in 96-well plates. Figure 1E shows the total green object integrated intensity (GCU × µm^2^/Image) in three cell groups (transfection group, negative control group, and blank group, n = 5 wells per group). As a result, the green fluorescence intensity increased with time which reached its peak at 96 h post-transfection. There were no statistical differences between the transfection group (LV) and the negative control group (LV-NC). In addition, the green fluorescence intensity in the blank group (NC) without transfection was maintained at a very low level at different time points (*p*-value < 0.0001). To visualize the cell transfection and fluorescent expression, two short videos for the LV group and LV-NC group at different time points after transfection were shown (Videos S1 and S2). Next, qRT-PCR was used to detect the expression of tiRNA-Gly-GCC-1 in the LV group and LV-NC group at 96 h post-transfection (n = 3 wells per group). The results showed that tiRNA-Gly-GCC-1 both in T24 and 5637 cells were significantly down-regulated after transfection. Compared to LV-NC, the relative expression level was lower in the LV group with significance (Figure 1F). These results altogether indicated that tiRNA-Gly-GCC-1 was inhibited successfully after lentivirus transfection.

### 3.2. Promotion of tiRNA-Gly-GCC-1 on Cell Proliferation and Confluence

**Figure 2:**
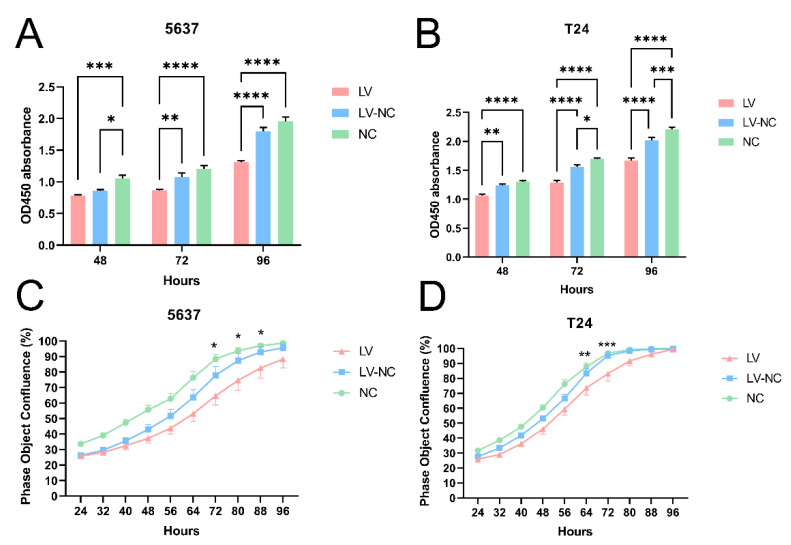
TiRNA-Gly-GCC-1 could promote cell proliferation and confluence (transfection group, LV; negative control group, LV-NC; blank group, NC). (**A**) CCK-8 assay: OD450 absorbance in 5637 cells. (**B**) CCK-8 assay: OD450 absorbance in T24 cells. (**C**) Cell confluence in 5637 cells. (**D**) Cell confluence in T24 cells. * indicates *p*-value < 0.05. ** indicates *p*-value < 0.01. *** indicates *p*-value < 0.001. **** indicates *p*-value < 0.0001.

To identify the biological function of tiRNA-Gly-GCC-1 on UBC cell lines, CCK-8 was used after the loss-of-function assay of tiRNA-Gly-GCC-1. As shown in Figure 1C, at 48 h, 72 h, and 96 h post-transfection, the transfection effect was significant. Thus, we observed the CCK-8 assay in three cell groups (transfection group, negative control group, and blank group, n = 5 wells per group) at these time points. As a result, in both 5637 and T24 cell lines (Figure 2A,B), down-regulation of tiRNA-Gly-GCC-1 repressed cell proliferation at 48 h, 72 h, and 96 h post-transfection. Compared to the LV group, the OD450 absorbance was higher in LV-NC and NC groups, especially at 72 h and 96 h post-transfection. However, there were statistical significances between LV-NC and NC groups at 48 h and 72 h post-transfection (*p*-value < 0.05). Besides, we used the live cell imaging system to observe the cell confluence in each group at 24–96 h post-transfection for 5637 and T24 cell lines. As shown in Figure 2C,D, down-regulation of tiRNA-Gly-GCC-1 delayed cell confluence after cell transfection. The cell confluence was higher in LV-NC and NC groups than in the LV group at each time point. There were statistical significances between LV-NC and NC groups at 72 h, 80 h, and 88 h in the 5637 cell line, while in the T24 cell line, the time points were 64 h and 72 h.

### 3.3. Promotion of tiRNA-Gly-GCC-1 on Cell Invasion and Migration

**Figure 3:**
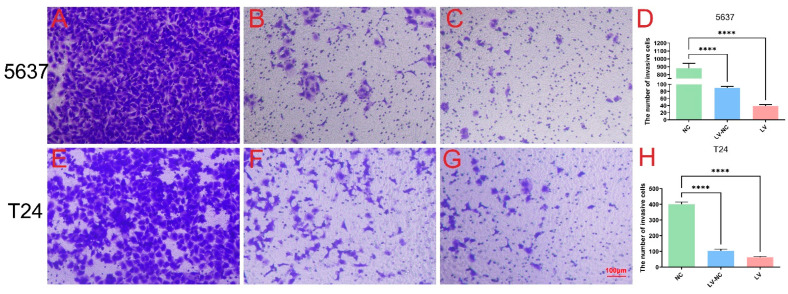
The Transwell invasion assay of three groups (transfection group, LV; negative control group, LV-NC; blank group, NC) in 5637 cells (**A**–**D**) and T24 cells (**E**–**H**). **** indicates *p*-value < 0.0001.

**Figure 4:**
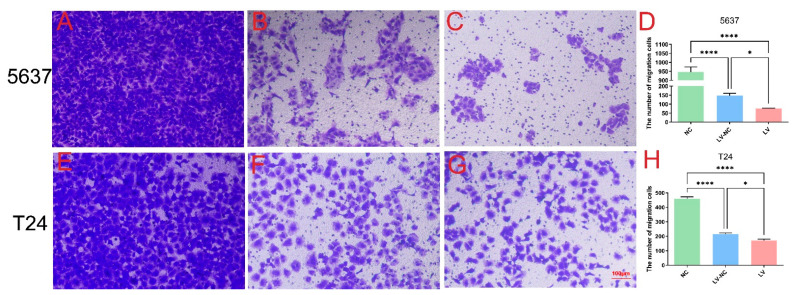
The Transwell migration assay of three groups (transfection group, LV; negative control group, LV-NC; blank group, NC) in 5637 cells (**A**–**D**) and T24 cells (**E**–**H**). * indicates *p*-value < 0.05. **** indicates *p*-value < 0.0001.

**Figure 5:**
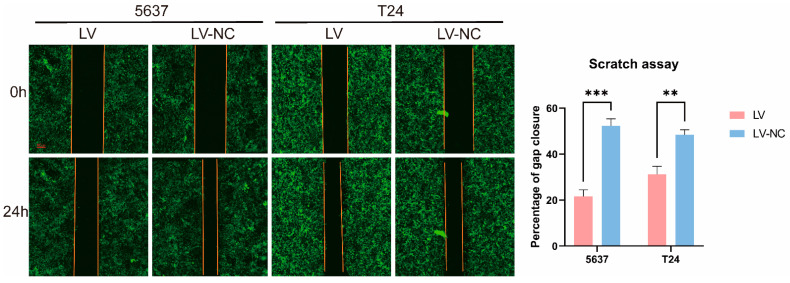
The scrape motility assay of two groups in 5637 and T24 cell lines (transfection group, LV; negative control group, LV-NC). ** indicates *p*-value < 0.01. *** indicates *p*-value < 0.001.

The effects of tiRNA-Gly-GCC-1 on cell invasion and migration were identified by Transwell and scrape motility assays. In terms of the Transwell invasion assay (Figure 3), the results showed that both in 5637 cells (Figure 3A–D) and T24 cells (Figure 3E–H), the number of invasive cells was lower in the LV group than in the LV-NC group. This indicated down-regulation of tiRNA-Gly-GCC-1 could repress the cell invasion. However, the cell invasion ability of LV and LV-NC groups was diminished compared to the NC group. In addition, it was speculated that lentivirus transfection affected the invasion ability of the cells. In terms of the Transwell migration assay (Figure 4), the results showed that both in 5637 cells (Figure 4A–D) and T24 cells (Figure 4E–H), the number of migrative cells was lower in the LV group than in the LV-NC group. This indicated down-regulation of tiRNA-Gly-GCC-1 could repress the cell migration. However, the cell migration ability of LV and LV-NC groups was diminished compared to the NC group. Moreover, it was speculated that lentivirus transfection affected the migration ability of the cells. Then, scrape motility assays showed that the percentage of gap closure was lower in the LV group compared to the LV-NC group. This indicated that cell motility was reduced after tiRNA-Gly-GCC-1 knock down in 5637 and T24 cell lines (Figure 5). To visualize the scrape motility of UBC cells, two short videos for the LV group (Video S3) and LV-NC group (Video S4) from day 0 to day 3 after transfection were offered.

### 3.4. Regulation of tiRNA-Gly-GCC-1 on Cell Cycle and Apoptosis

**Figure 6:**
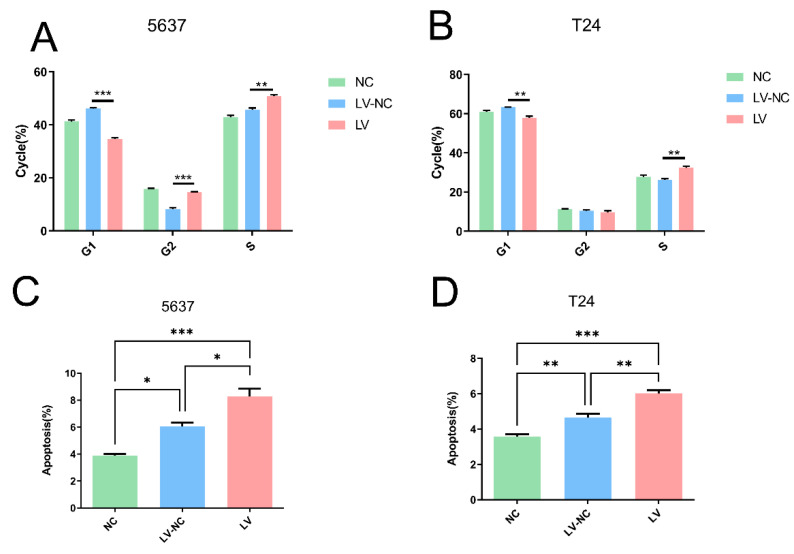
The cell cycle and apoptosis assays detected by flow cytometry in three groups (transfection group, LV; negative control group, LV-NC; blank group, NC). (**A**) Cell cycle of 5637 cells. (**B**) Cell cycle of T24 cells. (**C**) Cell apoptosis of 5637 cells. (**D**) Cell apoptosis of T24 cells. * indicates *p*-value < 0.05, ** indicates *p*-value < 0.01, *** indicates *p*-value < 0.001.

In this part, flow cytometry was used to explore the potential regulation of tiRNA-Gly-GCC-1 on cell cycle and apoptosis in 5637 and T24 cell lines. As shown in Figure 6A, 5637 cells in the LV group had a lower G1 phase, higher G2 phase, and higher S phase with statistical significance compared to the LV-NC group. As shown in Figure 6B, T24 cells in the LV group had a lower G1 phase, higher G2 phase, and higher S phase with statistical significance compared to the LV-NC group. These results altogether indicated that down-regulation of tiRNA-Gly-GCC-1 could affect the cell cycle and delay the synthesis of cellular DNA and proteins, suggesting a decreased cell proliferation rate. In terms of cell apoptosis of 5637 (Figure 6C) and T24 cells (Figure 6D), apoptosis rate (UR+LR) was elevated in the LV group compared to the LV-NC group with statistical significance. These results indicated that down-regulation of tiRNA-Gly-GCC-1 could promote cell apoptosis. However, after cell transfection, the apoptosis rate was higher in LV and LV-NC groups compared to the NC group. It was speculated that lentivirus transfection could affect cell viability due to cytotoxicity. The representative images for cell cycle and apoptosis were offered in Figures S1 and S2.

### 3.5. The Relationship of tiRNA-Gly-GCC-1 and TLR4

**Figure 7:**
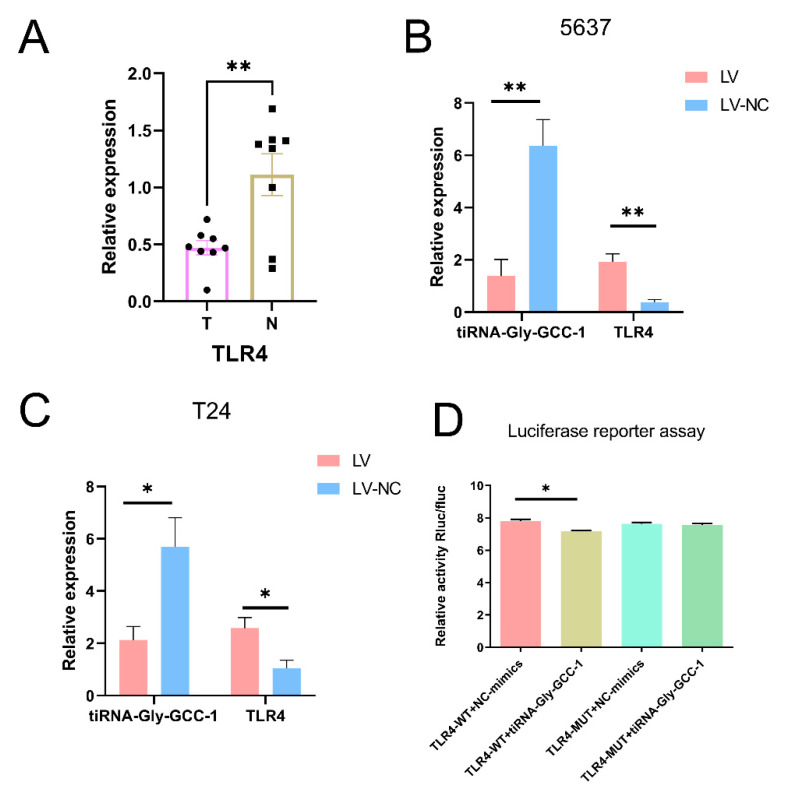
The relationship between tiRNA-Gly-GCC-1 and TLR4 (transfection group, LV; negative control group, LV-NC; tumor group, T; paracancerous tissues group, N). The data were normalized using the mean ± SEM. (**A**) The relative expression of TLR4 in UBC and paracancerous tissues. (**B**) The relative expression of two genes in LV and LV-NC group in 5637 cells. (**C**) The relative expression of two genes in LV and LV-NC group in T24 cells. (**D**) The luciferase assay for tiRNA-Gly-GCC-1 and TLR4. * indicates *p*-value < 0.05. ** indicates *p*-value < 0.01.

In our previous study [7], we found that tiRNA-Gly-GCC-1 was significantly upregulated in UBC tissues compared to paracancerous tissues, which might target TLR4 via base complementary pairing using bioinformatics. TLR4 might act as a potential target gene of tiRNA-Gly-GCC-1. The binding sites were shown in Figure S3. To identify their relationship, we first performed the qRT-PCR in UBC and paracancerous tissues for TLR4. As shown in Figure 7A, compared to paracancerous tissues (N), TLR4 (*p*-value < 0.01) was significantly down-regulated in tumor tissues (T). Thus, there was an opposite expression pattern for tiRNA-Gly-GCC-1 and TLR4. Next, qRT-PCR was used to detect the expression of tiRNA-Gly-GCC-1 and TLR4 in 5637 and T24 cell lines after cell transfection (LV and LV-NC group). As shown in Figure 7B,C, the results showed that compared to LV-NC, in the 5637 and T24 cell lines, tiRNA-Gly-GCC-1 was down-regulated while TLR4 was upregulated significantly in the LV group (*p*-value < 0.05). These results suggested an opposite change of expression of tiRNA-Gly-GCC-1 and TLR4. Last, a luciferase reporter assay was conducted to confirm the direct binding relationship between the two genes. In addition, as seen in Figure 7D, the luciferase activity of wild-type TLR4 3′UTR significantly decreased in the tiRNA-Gly-GCC-1 group compared to the corresponding NC mimics group, while the luciferase activity of mutant TLR4 3′UTR had no statistical significances between the tiRNA-Gly-GCC-1 group and corresponding NC mimics group. These results altogether indicated that the TLR4 expression was inhibited by the tiRNA-Gly-GCC-1 directly targeting its 3′UTR.

## 4. Discussion

Here, we found that tiRNA-Gly-GCC-1, upregulated in UBC tissues, could enhance cell proliferation, migration and invasion, inhibit cell apoptosis, and affect the cell cycle in UBC cell lines using various experiments of cellular and molecular biology. Besides, we verified that tiRNA-Gly-GCC-1 could inhibit TLR4 expression by directly targeting its 3′UTR, which was predicted as a target gene previously. Thus, the results showed that tiRNA-Gly-GCC-1 was closely related to the pathophysiological processes of UBC. To date, the present study offers the functional verifications of certain tsRNA in UBC cell lines for the first time. UBC develops following a stepwise accumulation of multiple genetic and epigenetic changes with complicated regulatory interaction networks during tumor initiation and progression. However, the precise molecular regulatory mechanisms are not yet fully understood. In this study, the biological functions of tiRNA-Gly-GCC-1 were identified to provide a new theoretical basis for exploring the molecular mechanism of UBC progression and great possibilities for further investigation.

In fact, tsRNAs were initially detected in the urine of patients with cancer in 1977 [15]. In the past, tsRNAs were originally considered as “turnover of tRNAs” in tumor tissues. It was not until 2009 that the mechanism of tsRNAs was firstly revealed [16]. Some studies have reported tsRNAs are dysregulated and play important roles in various diseases. For instance, tRFs derived from tRNALysCTT and tRNAPheGAA were found to act as a good indicator of progression-free survival and a candidate prognostic marker in prostate cancer [17]. It was reported that tsRNA-16902 was able to regμLate hMSC adipogenic differentiation by targeting RARγ via the Smad2/3 signaling pathway [18]. In clear cell renal cell carcinoma, 5′-tRNA-Arg-CCT, 5′-tRNA-Glu-CTC, and 5′-tRNA-Lys-TTT halves were circμLating at lower levels in serum samples of patients, indicating a potential role as a tumor suppressor [19]. Some researchers offered a comprehensive catalog of tRFs involved in papillary thyroid cancer and assessed the abnormal expression of these fragments [20].

After the literature review, we found only three studies related to tsRNA and UBC were reported. The researchers used bioinformatics to process The Cancer Genome Atlas (TCGA) data and collected UBC tissue samples for PCR validation. They found that the expression of 5′-TRF-LySCtt in UBC tissue was positively correlated with the progression rate of UBC (multivariate Cox: HR = 2.368; *p*-value = 0.033) and negatively correlated with the prognosis of patients (multivariate Cox: HR = 2.151; *p*-value = 0.032) [21]. Another study found abundant tsRNAs in UBC which contained modifications that can be captured as a mismatch by TGIRT sequencing [22]. Lastly, TRMT6/61A-dependent base methylation of tsRNAs was stated to regμLate gene-silencing activity and the unfolded protein response in UBC [23]. However, functional studies of tsRNA are lacking to date. In the present study, in UBC cell lines, we identified the functional roles of tiRNA-Gly-GCC-1 after gene knockdown. It is suggested that tiRNA-Gly-GCC-1, upregulated in UBC tissues, could act as an oncogene to promote the progression of UBC. Together with other tsRNAs studies focusing on UBC, the results indicated that specific tsRNAs could not only serve as biomarkers for UBC detection or follow-up but also be involved in the pathophysiology of UBC progression.

In terms of functional mechanism of tsRNAs, there were the following ways of tsRNA regulation [10]: (i) Formation of RNA-induced silencing complex by binding AGO proteins followed by targeting mRNA and inhibiting its expression for gene silencing at the post-transcriptional level; (ii) regulation of protein translation, namely, inhibition of the initiation and prolongation of the translation process; (iii) regulation of various intracellular activities; (iv) immune mediation and stress response. Among them, a lot of researchers have focused on the first downstream mechanism in which the tsRNA–mRNA signaling axis is involved in the pathophysiological changes of diseases [24]. For example, tRNALeu-derived tRF promoted cancer cell proliferation and invasion by inhibiting JAG2 in a miRNA-like manner in colorectal cancer cell lines [25]. In a study of ovarian cancer, tRF5-Glu inhibited cancer cell proliferation by binding to the repressive target gene BCAR3 [11]. In a study of small cell lung cancer, overexpression of tRF-Leu-CAG was detected in patients’ cancer tissues, cell lines, and serum. Then, silencing this gene could inhibit cancer cell proliferation and change the cell cycle by regulating photokinase A [26]. In our previous study [7], we found that tiRNA-Gly-GCC-1 could recognize TLR4 targets using their seed sequence and were predicted to have a close relationship with TLR4. Therefore, we identified their relationship in this study. Using PCR and luciferase reporter assay, tiRNA-Gly-GCC-1 was verified to directly target TLR4 and inhibit the expression of TLR4. Thus, in terms of the mode of action of the TLR4 target and tiRNA-Gly-GCC-1, we reasonably speculated that tiRNA-Gly-GCC-1 might bind the target TLR4 as a “sponge” through an RNA-induced silencing complex. Namely, tiRNA-Gly-GCC-1 could function as a competitive endogenous RNA (ceRNA) to suppress target TLR4. However, expression of the AGO protein should be detected to verify this speculation in our next study. Toll-like receptors (TLRs) agonists are considered effective immunostimulants with immunotherapeutic potential against several cancers including UBC. Bacillus Calmette–Guerin (BCG) (agonist of TLR2 and TLR4) is approved by The Food and Drug Administration (FDA) for intravesical UBC treatment [27]. Some researchers found TLR4 was down-regulated in UBC tissue compared to normal tissue and surrounding tumor. TLR4 expression can effectively predict oncological outcomes and drug sensitivity of BLCA patients. TLR4 was also associated with infiltrating immune cell variation and cancer pathway dysregulation [28]. In another study, researchers found that TLR4 may contribute to immune escape of UBC. The above results were in line with our findings in this study [29]. These results help us to perform the “rescue” experiments and explore the signal pathways of tiRNA-Gly-GCC-1 in the future.

However, this study has several limitations. First, this study explored the functional roles of tiRNA-Gly-GCC-1 and identified the relationship with the target gene TLR4 in UBC cell lines. In the future, “rescue” experiments should be conducted to verify the tiRNA-Gly-GCC-1/TLR4 pathways. Next, it is necessary to expand the UBC tissue samples to explore the possibilities of tiRNA-Gly-GCC-1 as a biomarker for UBC detection or follow-up. Besides, in terms of the relationship between tiRNA-Gly-GCC-1 and TLR4, the protein level of TLR4 should also be detected. Last but not least, the results of this study showed that lentivirus had an adverse effect on the activity of UBC cells. Thus, other transfection reagents such as adenovirus and adeno-associated virus could be attempted.

## 5. Conclusions

In summary, tiRNA-Gly-GCC-1 could promote tumor progression and inhibit TLR4 expression by directly targeting its 3′UTR. This study provides novel insights for future investigations to explore the mechanisms and therapeutic targets for UBC.

## Data Availability

The data presented in this study are available on request from the corresponding author. The data are not publicly available due to institutional policy.

## Supplementary Materials

The following are available online at https://www.mdpi.com/article/10.3390/cancers14194555/s1, Figure S1: The representative images for cell apoptosis; Figure S2: The representative images for cell cycle; Figure S3: The 2D structure and binding sites predicted by bioinformatics for TLR4 and tiRNA-Gly-GCC-1; Table S1: List of sequences used in this study; Video S1: The visualization of the cell transfection and fluorescent expression in transfection group (LV); Video S2: The visualization of the cell transfection and fluorescent expression in negative control group (LV-NC); Video S3: The visualization of the scrape motility of UBC cells in transfection group (LV); Video S4: The visualization of the scrape motility of UBC cells in negative control group (LV-NC).

## Abbreviations

BC: bladder cancer; UBC: urothelial bladder carcinoma; MIBC: muscle-invasive bladder cancer; NMIBC: non-muscle-invasive bladder cancer; ncRNAs: noncoding RNAs; miRNAs: microRNAs; tRNAs: transfer RNAs; tsRNA: tRNA-derived small RNA; mRNAs: messenger RNAs; tRFs: tRNA-derived fragments; tiRNAs: tRNA-derived stress induced RNAs; qRT-PCR: quantitative reverse transcription-polymerase chain reaction; SEM: standard error of the mean; TLR4: toll-like receptor 4; DMEM: DμLbecco’s Modified Eagle’s Medium; GFP: green fluorescent protein; CCK-8: Cell Counting Kit-8; TCGA: The Cancer Genome Atlas; GAPDH: glyceraldehyde-3-phosphate dehydrogenase; BCG: Bacillus Calmette–Guerin; FDA: The Food and Drug Administration.
