# Supplementary material for: A Novel tiRNA-Gly-GCC-1 Promotes Progression of Urothelial Bladder Carcinoma and Directly Targets TLR4
Source: Cancers (Basel). 2022 Sep 20;14(19):4555. doi: 10.3390/cancers14194555 (PMC9558499; doi:10.3390/cancers14194555)
Supplement: Supplementary file 1 [file cancers-14-04555-s001.zip › Supplementary_Material - 20220808.pdf]

cancers

MDPI

Supplementary Material

A novel tiRNA-Gly-GCC-1 promotes progression of urothelial bladder carcinoma and directly targets TLR4

Chuan Qin[1], Zheng-Hao Chen[1], Rui-Cao[1], Ming-Jun Shi[1], Ye Tian[1,*]

*** Correspondence:**
Dr. Ye Tian, youyitianye@126.com.

Table S1. List of sequences used in this study.

Name	Sequence (5' - 3')
ShRNA target	CAGGCGAGAATTCTACCACTGAACCACCCATGC
Negative control shRNA	TTCTCCGAACGTGTCACGT
TiRNA-Gly-GCC-1 Forward	TACAGTCCGACGATCGCATG
TiRNA-Gly-GCC-1 Reverse	CGTGTGCTCTTCCGATCTCA
TLR4 Forward	TCCCTCCAGGTTCTTGATTACAG
TLR4 Reverse	GCCCTGCTTATCTGAAGGTGTT
GAPDH Forward	GGAAGCTTGTCATCAATGGAAATC
GAPDH Reverse	TGATGACCCTTTTGGCTCCC

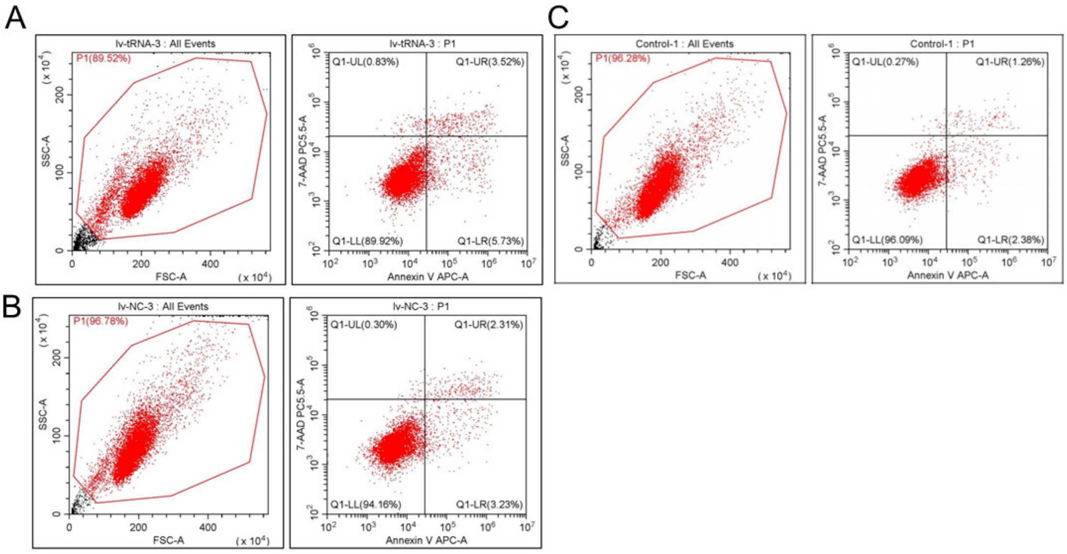

Figure S1. The representative images for cell apoptosis. (A) transfection group (LV); (B) negative control group (LV-NC); (C) blank group (NC). Lower left quadrant (LL) stands for live cells; lower right quadrant (LR) stands for early apoptotic cells, upper right quadrant (UR) stands for late apoptotic and dead cells; upper left quadrant (UL) limit is debris and damaged cells. The apoptosis rate can be calculated as UR+LR.

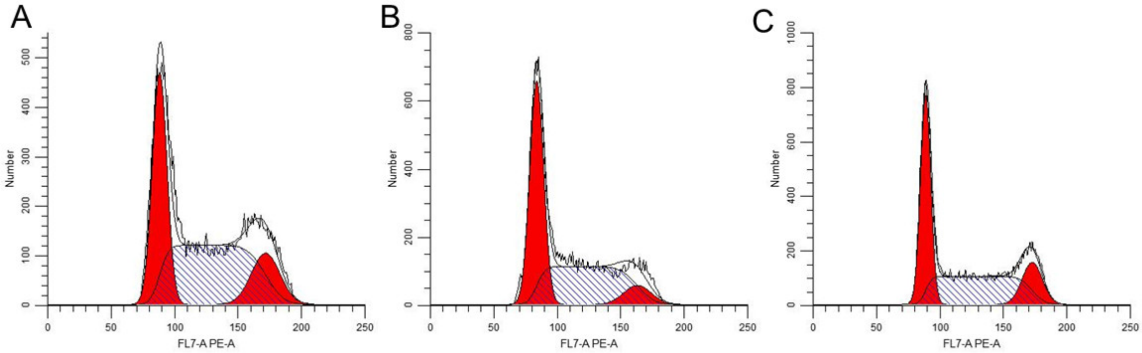

Figure S2. The representative images for cell cycle. (A) transfection group (LV); (B) negative control group (LV-NC); (C) blank group (NC). The first peak stands for the G1 phase. The second peak stands for the G1 phase. The wide peak between G1 and G2 stands for the S phase.

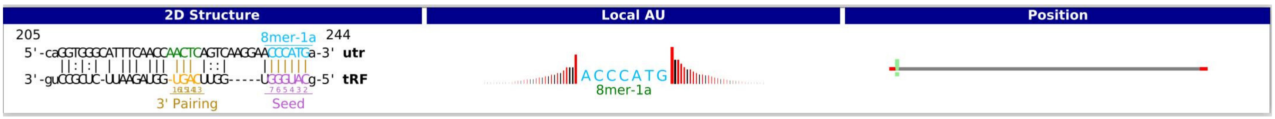

Figure S3. The 2D structure and binding sites predicted by bioinformatics for TLR4 and tiRNA-Gly-GCC-1.

Video S1. The visualization of the cell transfection and fluorescent expression in transfection group (LV) at 24h (0d) to 96h (3d) post transfection.

Video S2. The visualization of the cell transfection and fluorescent expression in negative control group (LV-NC) at 24h (0d) to 96h (3d) post transfection.

Video S3. The visualization of the scrape motility of UBC cells in transfection group (LV).

Video S4. The visualization of the scrape motility of UBC cells in negative control group (LV-NC).
